# Combining Regional and Connectivity Metrics of Functional Magnetic Resonance Imaging and Diffusion Tensor Imaging for Individualized Prediction of Pain Sensitivity

**DOI:** 10.3389/fnmol.2022.844146

**Published:** 2022-03-15

**Authors:** Rushi Zou, Linling Li, Li Zhang, Gan Huang, Zhen Liang, Lizu Xiao, Zhiguo Zhang

**Affiliations:** ^1^School of Biomedical Engineering, Health Science Center, Shenzhen University, Shenzhen, China; ^2^Guangdong Provincial Key Laboratory of Biomedical Measurements and Ultrasound Imaging, Shenzhen University, Shenzhen, China; ^3^Marshall Laboratory of Biomedical Engineering, Shenzhen, China; ^4^Department of Pain Medicine and Shenzhen Municipal Key Laboratory for Pain Medicine, The Affiliated Shenzhen Sixth Hospital of Guangdong Medical University, Shenzhen, China; ^5^Peng Cheng Laboratory, Shenzhen, China

**Keywords:** pain sensitivity, fMRI, DTI, regional-connectivity features, machine learning

## Abstract

Characterization and prediction of individual difference of pain sensitivity are of great importance in clinical practice. MRI techniques, such as functional magnetic resonance imaging (fMRI) and diffusion tensor imaging (DTI), have been popularly used to predict an individual’s pain sensitivity, but existing studies are limited by using one single imaging modality (fMRI or DTI) and/or using one type of metrics (regional or connectivity features). As a result, pain-relevant information in MRI has not been fully revealed and the associations among different imaging modalities and different features have not been fully explored for elucidating pain sensitivity. In this study, we investigated the predictive capability of multi-features (regional and connectivity metrics) of multimodal MRI (fMRI and DTI) in the prediction of pain sensitivity using data from 210 healthy subjects. We found that fusing fMRI-DTI and regional-connectivity features are capable of more accurately predicting an individual’s pain sensitivity than only using one type of feature or using one imaging modality. These results revealed rich information regarding individual pain sensitivity from the brain’s both structural and functional perspectives as well as from both regional and connectivity metrics. Hence, this study provided a more comprehensive characterization of the neural correlates of individual pain sensitivity, which holds a great potential for clinical pain management.

## Introduction

Pain is a subjective, complex, and multidimensional sensory experience that exhibits huge inter-subject variability (Rainville, 2002; Nielsen et al., 2009; Coghill, 2010). The study of individual differences in pain sensitivity is of great importance in clinical practice (Werner et al., 2010; Abrishami et al., 2011) and in pharmaceutical research (Chizh et al., 2009; Angst et al., 2012). For example, pain sensitivity is a predictive factor for the treatment outcome of many clinical diseases (Abrishami et al., 2011; Rehberg et al., 2017). Hence, investigating the underlying neural mechanism of individual differences in pain sensitivity cannot only deepen our understanding of pain sensitivity but can also be used to develop a predictive model of individual pain sensitivity.

With the fast development of neuroimaging technologies and associated data analytics, using neural images and signals, such as magnetic resonance imaging (MRI) and electroencephalography (EEG), to probe the neural mechanisms of pain has been widely adopted in pain researches, which include the studies of momentary (acute or chronic) pain experience (Apkarian et al., 2005) and pain sensitivity (Coghill et al., 2003; Zunhammer et al., 2016). The complex brain activity underlying pain sensitivity plays a major role in the representation and modulation of pain (Rainville, 2002; Apkarian et al., 2005). Several studies have revealed that individual differences in pain sensitivity are reflected in differences in brain structure and function by using different MRI modalities (Geisler et al., 2021; Niddam et al., 2021).

Resting-state functional magnetic resonance imaging (rs-fMRI) uses blood oxygenation level-dependent (BOLD) responses to study spontaneous brain activity in individuals when performing no specific task. A very widely used rs-fMRI feature is Regional Homogeneity (ReHo), which calculates Kendall’s coefficient of concordance to measure regional synchronizations of temporal changes in BOLD activities in a voxel-wise manner (Baumgartner et al., 1999). Several studies used ReHo to investigate the local features of spontaneous brain activity in chronic pain such as migraine (Yu et al., 2012; Zhao et al., 2013; Zhang et al., 2016) and headache (Wang et al., 2014). These ReHo-based results showed that patients with chronic pain exhibited increased or decreased ReHo values in certain regions compared to healthy subjects. For example, Yoshino et al. (2017) found that ReHo at the dorsolateral prefrontal cortex significantly decreased in chronic pain patients. Another type of rs-fMRI feature popularly used in pain research is functional connectivity (FC). Unlike ReHo, which measures regional brain activities, FC measures the statistical relationship between BOLD signals of different brain regions. Many studies have demonstrated that FC between some specific regions is related to pain perception and can be used as a neural indicator of individual pain sensitivity. For example, Tu et al. (2019) used multivariate pattern analysis to find that resting-state FC could predict individual pain thresholds with high accuracy (a correlation coefficient of 0.60 between predicted and real values of heat pain thresholds). Meanwhile, they found that the connections within medial-frontal and frontal-parietal networks are the most predictive FC features of pain sensitivity. Another study (Spisak et al., 2020) also identified and validated a pain-free resting-state FC pattern that is predictive of individual differences in pain sensitivity.

Besides rs-fMRI, diffusion tensor imaging (DTI), which maps white matter anatomical connections in the living human brain, is another common MRI modality that has been gradually used to study individual differences in pain sensitivity (Deppe et al., 2013; Porpora et al., 2018). Fractional anisotropy (FA) is the most widely used quantitative DTI measure and it reflects how the diffusion of water is directionally constrained along axons (Alexander et al., 2007). Several DTI studies have found abnormal white matter changes in migraine and other chronic pain conditions (Mansour et al., 2013; Michels et al., 2017). On the other hand, DTI is able to characterize the structural connectivity (SC) based on the fibers connecting each pair of brains regions. DTI-based SC has also been used to investigate the pain-related brain networks. For example, by using the graph analysis of probabilistic tractography based on DTI, one study found that the anterior insula connectivity was related to the individual degree of pain vigilance and awareness (Wiech et al., 2014). Also, studies demonstrated that SC provides new insights into the understanding of chronic pain. For example, Huang et al. (2021) found that patients with chronic prostatitis/chronic pelvic pain syndrome had alterations of SC within the frontal-parietal control network.

However, most of the existing MRI studies regarding pain sensitivity are limited by only using one single modality of MRI (rs-fMRI or DTI) or only using one single type of feature (regional or connectivity features) to explore the relationship between pain sensitivity and MRI features. However, pain has a complicated neural mechanism, which influences and is influenced by the brain’s structure and function. Also, both brain patterns within local regions and brain connections among local regions contribute to an individual’s sensitivity of pain. Thus, only using one MRI modality or using one type of feature (regional or connectivity) cannot offer a complete characterization of brain patterns related to pain and cannot provide sufficient information to accurately predict the individual pain sensitivity. Accumulated evidence have shown the importance of using multiple MRI modality in the understanding of cognitive functions and the diagnosis of neurological diseases (Michels et al., 2017; Dhamala et al., 2020). For example, Xiao et al. (2021) built a model to predict visual working memory capacity by using voxel-wise multimodal MRI features (amplitude of low-frequency fluctuations from fMRI, gray matter volume from structural MRI, and FA from DTI). On the other hand, MRI studies based on both regional patterns and inter-regional connectivity patterns are also gaining popularity in the research of brain disorders. For example, Luo et al. (2020) used multi-features, including both regional features and connectivity features extracted from fMRI and DTI, to significantly improve the prediction performance of adult outcomes in childhood-onset attention-deficit/hyperactivity disorder compared to using the models based on one type of features. However, combining both regional and connectivity features from both rs-fMRI and DTI in the prediction of pain sensitivity is still lacking. As a result, it remains unclear how the brain’s structure, function, and connectivity interact and synergize in the determination of an individual’s pain sensitivity.

In the present study, we hypothesize that both regional and connectivity features from both rs-fMRI and DTI are predictive of an individual’s pain sensitivity. This hypothesis was proposed based on the following facts. First, scattered evidence has shown that, either regional or connectivity patterns measured from either fMRI or DTI were correlated with an individual’s pain threshold (Rogachov et al., 2016; Hsiao et al., 2020; Geisler et al., 2021). Second, for either fMRI or DTI, its regional patterns and connectivity patterns are associated (Ma et al., 2010; Straathof et al., 2019). Third, because of the brain’s structural-functional coupling, fMRI and DTI are also correlated in terms of regional characteristics or connections (Gu et al., 2015; Tang and Bassett, 2018; Straathof et al., 2019). These literature supports will be further elaborated in the Discussion.

To validate this hypothesis, we acquired rs-fMRI and DTI data as well as laser pain threshold from 210 healthy participants and explored the relationship between multi-modal MRI features and individual pain sensitivity. For each participant, we extracted regional and connectivity features from two MRI modalities (ReHo and FC from rs-fMRI; FA and SC from DTI). We used machine learning and feature selection methods to construct prediction models and to identify the most predictive features of pain sensitivity. Furthermore, to examine whether different MRI modalities and different feature types can provide complementary information in predicting an individual’s pain thresholds, we established a series of models to fuse various types of MRI features at the decision level and compared their performance.

## Materials and Methods

### Participants

We recruited a total of 210 healthy participants (131 females; age: 20.81 ± 2.93 years) through college and community advertisements and paid for their participation. All the participants were right-handed. Before the experiments, participants were carefully screened to ensure that they had no history of chronic pain, neurological diseases, cerebrovascular diseases, coronary heart disease, and mental disorders, and they had no contraindications to MRI examination. The study was proved by the local ethics committee and all participants gave their written informed consent before participating in the study.

### Measurement of Pain Threshold

Pain sensitivity of all the participants was measured as the laser pain threshold in a behavioral experiment before the MRI scan. The laser pain threshold was measured manually using quantitative sensory testing. A series of infrared neodymium yttrium aluminum perovskite (Nd: YAP) laser stimuli were delivered to the back area between the thumb and index finger of a participant’s left hand. The measurement started from an energy level at 1 J with a 0.25 J increase at each stimulus. After each stimulus, a participant was asked to report the pain rating from 0 (no pain) to 10 (the worst pain). When a rating of 4 was reported, the corresponding energy level was recorded as the laser pain threshold. For each participant, the laser pain threshold was averaged from two independent measurements conducted in 1 h.

### Magnetic Resonance Imaging Acquisition

Multimodal MRI data were acquired using a GE 3.0 T scanner. Resting-state fMRI were collected using the following parameters: 43 oblique slices, thickness/gap = 3/0 mm, acquisition matrix = 64×64, TR = 2,000 ms, TE = 30 ms, flip angle = 90°, field of view = 22×22 mm^2^, total volume = 300, acquisition time = 10 min. For the DTI data, the following acquisition parameters were used: 70 axial slices, TR = 8,500 ms, TE = 80.8 ms, 64 optimal non-linear diffusion-weighted directions with b = 1,000 s/mm2 and one additional image without diffusion weighting (i.e., b = 0 s/mm2), 2.0-mm slice thickness, acquisition matrix = 128×128; 2×2 mm in-plane resolution, acquisition time = 10:50 min.

### Data Analysis

#### Functional Magnetic Resonance Imaging Preprocessing

Resting-state fMRI preprocessing was performed with DPABI^1^ (Yan et al., 2016) and SPM12 (Statistical Parametric Mapping; Wellcome Department of Imaging Neuroscience, University College London, United Kingdom)^2^ running under Matlab R2017b (Mathworks, Sherborn, MA). For each subject, the first 10 volumes of rs-fMRI data were discarded, leaving 290 images pre-processed. The middle slice was used as the reference slice for slice timing correction. Then, fMRI data were realigned to correct the head motion and obtained the 6 rigid body motion parameters. T1 images were co-registered to functional images and segmented into gray matter, white matter, and cerebrospinal fluid. In order to decrease the effects of head motion, the Friston 24-parameter model, 6 head motion parameters, 6 head motion parameters one time point before, and the 12 corresponding squared items (Friston et al., 1996), were used to regress out the head motion parameters. Time points with the head motion parameters larger than 0.2 were scrubbed, and they were modeled as a separate covariable for regression to decrease their influence on the continuity of time. The functional images were then normalized into standard Montreal Neurological Institute (MNI) space, resampled to a 3 × 3 × 3 mm^3^ voxel. Finally, a bandpass filter with a frequency window of 0.01–0.1 Hz was used to improve the signal-to-noise ratio of fMRI signals.

#### Diffusion Tensor Imaging Preprocessing

The DTI data were preprocessed by the PANDA toolbox (Cui et al., 2013)^3^ in the FSL diffusion toolkit and MRIcron. The preprocessing steps were performed as follows. Briefly, (a) covert the DICOM files of all subjects into NIfTI images using the MRIcron; (b) estimate the brain mask by extracting the brain tissue and structure; (c) correct for the eddy-current effect; (d) average acquisitions and calculate DTI metrics. In order to get the voxel-based diffusion metrics for the subsequent analysis, the individual diffusion metric images were transformed from the native space into a standard Montreal Neurological Institute (MNI) space (voxel size 1 mm × 1 mm × 1 mm^3^) via spatial normalization and smoothed with a 6 mm full width at half-maximum (FWHM) Gaussian kernel.

#### Brain Parcellation

In order to better compare the results of different imaging modalities, we used the Automated Anatomical Labeling (AAL) (Tzourio-Mazoyer et al., 2002) atlas, which was commonly adopted in multimodality researches to achieve the whole-brain parcellation on both functional data and structural data (Wee et al., 2012; Ahmed et al., 2017). Cerebellar regions were excluded for incomplete coverage of the cerebellum of several participants. In total 90 regions of interest (ROIs) were defined by the AAL atlas and used in subsequent analyses.

#### Feature Extraction

We extracted both regional and connectivity metrics from pre-processed fMRI and DTI for the prediction of the laser pain threshold.

*fMRI ReHo:* For fMRI, individual ReHo maps were generated by calculating Kendall’s coefficient concordance of the time series of a given voxel with those of its surrounding 27 voxels (Zang et al., 2004). Then, the data were smoothed with a Gaussian filter of 6 mm FWHM to reduce noise and residual differences in gyral anatomy. These individual maps underwent whole-brain equalization for further analysis. Finally, 55017 ReHo features were extracted for each participant.

*fMRI FC:* For fMRI FC matrices, Pearson’s correlation coefficients (PCC) between BOLD time courses of each pair of ROIs were calculated for each subject. The obtained correlation matrix for each subject was then normalized using Fisher’s z-transformation to improve normality. The FC matrix for each individual was a 90×90 symmetric matrix. Only the lower triangular matrix, which has 4005 FC features, was taken for subsequent analysis.

*DTI FA:* For DTI, the FA matrix, which measures the degree of anisotropy of water diffusion, was calculated in the MNI space for each individual. FA is calculated from the eigenvalues of the diffusion tensor, and its value varies between 0 and 1. FA = 0 means that the diffusion ellipsoid is a sphere (perfect isotropic diffusion). When the eigenvalues become more unequal with progressive diffusion anisotropy, the FA → 1. Finally, for each participant, 55017 FA features were extracted for the following analysis.

*DTI SC:* After the pre-processing of DTI data, probabilistic tractography was used to construct the SC network (Behrens et al., 2007). Briefly, for each defined brain region/node, probabilistic tractography was performed by seeding from all voxels of this region. For each voxel, 5000 fibers were sampled. The connectivity probability from the seed region *i* to another region *j* was defined by the number of fibers passing through region *j* divided by the total number of fibers sampled from region *i*. The connectivity probability of each node to the other nodes within the brain network can be calculated by repeating the tractography procedure for all nodes. This leads to an individual-specific weighted matrix, whose rows and columns represent the brain nodes and whose elements represent the connectivity probability between nodes. The SC matrix for each participant was a 90×90 matrix.

#### Prediction of Pain Threshold

**FIGURE 1:**
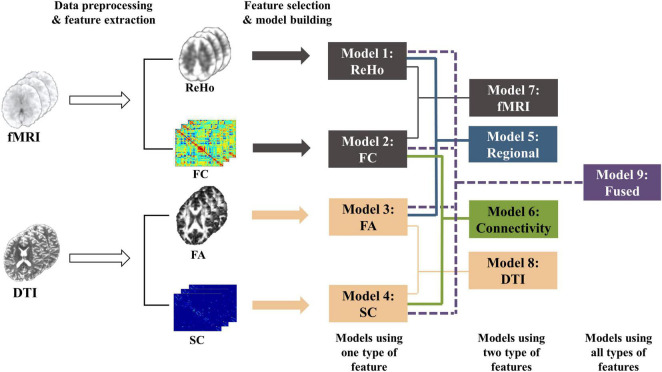
The whole procedure of the pain threshold prediction analysis based on multi-features of multi-modal MRI.

After regional features (i.e., ReHo and FA) and connectivity features (i.e., FC and SC) were extracted from fMRI and DTI data, we used feature selection and machine learning techniques to establish models for predicting individual laser pain thresholds. As shown in Figure 1, the whole procedure of pain threshold prediction is detailed as follows.

*Cross-validation:* We selected the features, trained and tested the prediction models based on the leave-one-individual-out cross-validation. At each run, we randomly used one participant’s data for testing and the remaining participants’ data for training. Because we had a total of 210 participants, the procedure was repeated 210 times to make sure that each participant’s data were used as test samples once.

*Feature selection:* To improve the model accuracy and increase the model interpretability, it is necessary to identify a subset of most predictive features from a high-dimensional feature. We adopt the correlation-based feature selection method for each type of feature (ReHo, FA, FC, or SC) separately. In each run of the cross-validation in regression, PCC between each type of feature and the laser pain thresholds were computed within the training data to make sure the test data were not involved in the step of feature selection. The features with correlation significance beyond a threshold (*P* = 0.05; *P* = 0.01; *P* = 0.001, tested separately for comparison, see Supplementary Tables 1, 2 for details) were selected and used for the prediction of pain thresholds.

*Machine learning algorithms:* After feature normalization, four popular and effective ML regression algorithms were used to model the relationship between these MRI features and laser pain threshold, namely, support vector machine regression with linear kernels (SVR-Linear) or Gaussian kernels (SVR-RBF), partial least squares regression (PLSR), and random forest regression (RF). All these algorithms were implemented with the open-source scikit-learn library for python (Pedregosa et al., 2011).

*Models with different feature types:* We compared the prediction performance of a series of models based on single-modality and single-type features using different machine learning algorithms and different thresholds of feature selection. Supplementary Tables 1, 2 show the prediction performance of these models. Four models using one type of feature, namely ReHo (SVR-Linear, threshold < 0.001), FA (SVR-RBF, threshold < 0.01), FC (SVR-Linear, threshold < 0.001), and SC (SVR-RBF, threshold < 0.001) models, were determined first because of their better performance than models with other ML algorithms and parameters. Then, features were selected based on above four models using the corresponding feature type. As a result, the fusion process allowed information from multiple modalities to integrate but did not interfere with the feature selection and model selection in each model (ReHo, FA, FC, or SC model). Next, we build five models using different combinations of features: ReHo + FA (regional features), FC + SC (connectivity features), ReHo + FC (fMRI features), FA + SC (DTI features), and all four features. An average of the predicted values of multiple single-type feature models was calculated as the final predicted threshold of each multimodality models. More precisely, we established and compared the following 9 models with different types of features:

1. ReHo Model: using ReHo features;
2. FC Model: using FC features;
3. FA Model: using FA features;
4. SC Model: using SC features;
5. Regional Model: using ReHo (from rs-fMRI) and FA (from DTI) features;
6. Connectivity Model: using FC (from rs-fMRI) and SC (from DTI) features;
7. fMRI Model: using ReHo (regional) and FC (connectivity) features from fMRI;
8. DTI Model: using FA (regional) and SC (connectivity) features from DTI;
9. Fused Model: using all features (ReHo + FA + FC + SC).

*Performance evaluation:* PCC between the predicted thresholds and the true values across all participants was calculated as the main metric of the performance of these prediction models. Also, we calculated the mean absolute error (MAE), which measured the overall distance between predicted and true values. MAE is calculated as:


$$MAE=\frac{1}{N}\sum_{n=1}^{N}\left|\hat{y_{n}}-y_{n}\right|,$$

where *y*_*n*_ is the measured pain threshold of the *n*-th participant, $\hat{y_{n}}$ is the pain threshold estimated from the prediction model, and *N* is the total number of participants. The prediction performance in terms of MAE of nine models was compared using paired *t*-test. The PCCs of any two models were compared using the test for comparing elements of a correlation matrix, as suggested in Steiger (1980). This correlation test was adopted here because the true labels were used in the calculation of all PCCs so that these PCCs were not independent and the conventional *t*-test could not be used.

*Identifying common predictive features:* This part is aimed at identifying the brain regions and brain connectivity that are commonly selected across individuals. We calculated the occurrence frequency of each feature across all folds in leave-one-individual-out cross-validation involved in building the models based on one type of feature. For better visualization and interpretation of the features, we only showed those features which were selected more than half of the time in the whole leave-one-individual-out cross-validation procedure. ReHo and FA results were mapped onto the AAL-90 atlas. Connectivity results were visualized by using the Connectivity Visualization Tool^4^.

## Results

### Measurements of Pain Threshold

For all participants, the laser pain thresholds were 2.57 ± 0.53 J (mean ± std). We calculated the PCC between age and pain sensitivity but found no significant relationship (*p* = 0.98) between age and laser pain threshold. A two-sample *t*-test revealed that gender had no significant effect on the laser pain threshold (*p* = 0.49).

### Prediction Performance of Different Models

**FIGURE 2:**
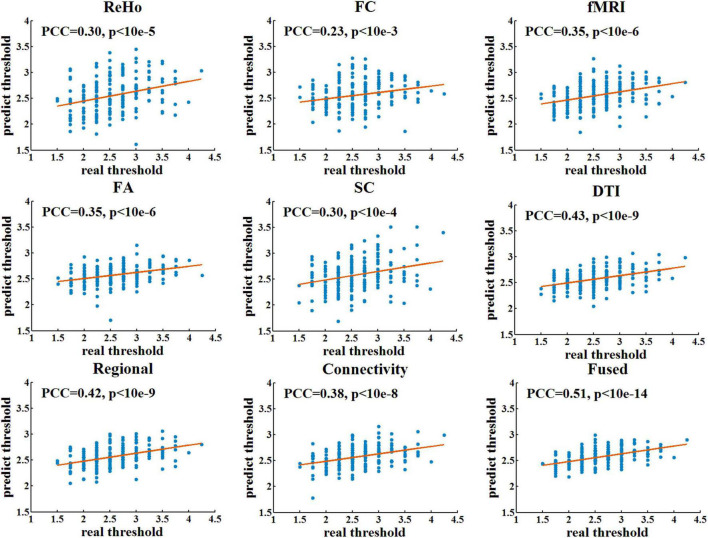
The linear correlation between predicted and real pain thresholds. Each blue dot denotes one participant. Red lines are linear fitting lines.

**FIGURE 3:**
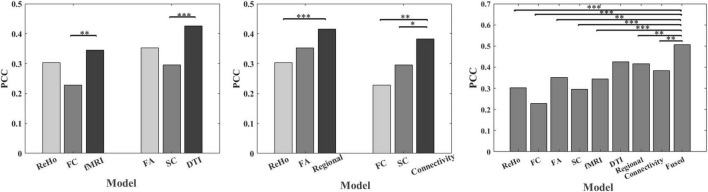
Comparison of PCC between predicted and real laser pain threshold among models using different features. * indicates *p* < 0.05, ^**^ indicates *p* < 0.01, ^***^ indicates *p* < 0.001.

Table 1 shows the prediction performance of nine laser pain threshold prediction models: ReHo Model (PCC = 0.30, *p* = 7.64×10^–6^), FC Model (PCC = 0.23, *p* = 8.73×10^–4^), FA Model (PCC = 0.35, *p* = 1.61×10^–7^), SC Model (PCC = 0.30, *p* = 1.36×10^–5^), fMRI Model (PCC = 0.35, *p* = 2.91×10^–7^), DTI Model (PCC = 0.43, *p* = 1.16×10^–10^), Regional Model (PCC = 0.42, *p* = 3.73×10^–10^), Connectivity Model (PCC = 0.38, *p* = 9.54×10^–9^), and Fused Model (PCC = 0.51, *p* = 4.99×10^–15^). As shown in Figure 2, the correlation results of all prediction models are significant. Figure 3 compares the PCC between predicted and real laser pain thresholds of all the participants among different prediction models. We have the following two major observations from Table 1 and Figures 2, 3. First, the prediction performances in terms of PCC of the models based on two type features (i.e., Regional Model, Connectivity Model, fMRI Model, and DTI Model) are higher than models which only used one type of feature. Specifically, the correlation result of fMRI Model is significantly better than FC Model (*p* = 0.007), and PCC of DTI Model is significantly better than SC model (*p* = 4.19×10^–5^). Also, PCC of Regional Model is significantly better than ReHo model (*p* = 6.90×10^–5^), and PCC of Connectivity Model is significantly better than FC model (*p* = 0.002) and SC model (*p* = 0.05). Second, the correlation result of Fused Model is significantly better than all models based on one-type (Fused vs. ReHo, *p* = 2.08×10^–5^; Fused vs. FC, *p* = 2.67×10^–7^; Fused vs. FA, *p* = 0.007; Fused vs. FT, *p* = 4.49×10^–4^) or two type features (Fused vs. fMRI, *p* = 3.43×10^–6^; Fused vs. DTI, *p* = 0.057; Fused vs. Regional, *p* = 0.011, Fused vs. Connectivity, *p* = 9.29×10^–4^).

**TABLE 1:** Prediction performance of different models using different feature sets.

Feature set	MAE (mean ± std)	PCC (R and *p*-values)
ReHo	0.42 ± 0.33	0.30 (7.64×10^–6^)
FC	0.43 ± 0.32	0.23 (8.73×10^–4^)
FA	0.39 ± 0.29	0.35 (1.61×10^–7^)
SC	0.41 ± 0.32	0.30 (1.36×10^–5^)
fMRI (ReHo + FC)	0.39 ± 0.31	0.35 (2.91×10^–7^)
DTI (FA + SC)	0.37 ± 0.30	0.43 (1.16×10^–10^)
Regional (ReHo + FA)	0.37 ± 0.30	0.42 (3.73×10^–10^)
Connectivity (FC + SC)	0.38 ± 0.30	0.38 (9.54×10^–9^)
Fused (ReHo + FC + FA + SC)	**0.36 ± 0.29**	**0.51** (4.99×10^–15^)

*Highlight the best performance of the prediction model.*

### Predictive Multimodality Features

**FIGURE 4:**
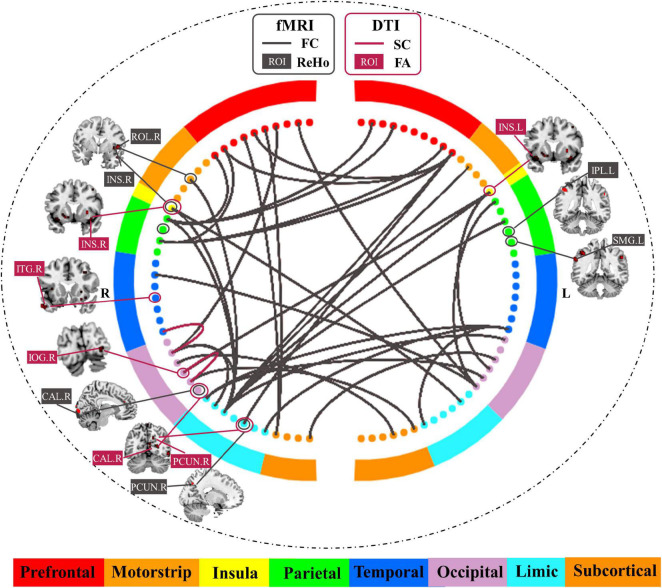
Common predictive regional features and connectivity features for the prediction of laser pain threshold.

Table 2 and Figure 4 show the common predictive regional feature sets (ReHo or FA, respectively), which were determined because they were selected for more than half of the time in the leave-one-individual-out cross-validation for the laser pain threshold prediction. Finally, ReHo features of 109 voxels were selected and they were mainly in Parietal_Inf_L, SupraMarginal_L/R, Insula_R, Rolandic_Oper_R, Calcarine_R, Temporal_Mid_R, Precuneus_R, Cingulum_Mid_R. FA features of 668 voxels were selected and they were mainly in Occipital_Inf_R, Temporal_Inf_R, Calcarine_R, Precuneus_R, Insula_L/R, Frontal_Mid_R, Temporal_Pole_Mid_L, Putamen_L/R, Lingual_R. The common regions of ReHo and FA feature sets are Precuneus_R, Insula_R, and Calcarine_R.

**TABLE 2:** List of common predictive regional features for the prediction of laser pain threshold.

Feature set	Regions or connectivity
ReHo	Parietal_Inf_L, SupraMarginal_L/R, Insula_R, Rolandic_Oper_R, Calcarine_R, Temporal_Mid_R, Precuneus_R, Cingulum_Mid_R
FA	Occipital_Inf_R, Temporal_Inf_R, Calcarine_R, Precuneus_R, Insula_L/R, Frontal_Mid_R, Temporal_Pole_Mid_L, Putamen_L/R, Lingual_R
Common regions	Insula_R, Calcarine_R, Precuneus_R

As shown in Table 3 and Figure 4, there were 35 common FC features and 2 common SC features were visualized because they were selected for more than half of the time in the leave-one-individual-out cross-validation. These FC features are predominately for the prefrontal-parietal and insula-cingulate networks. For SC, the 2 features are Occipital_Inf_R-Lingual_R, Fusiform_R-Occipital_Sup_R. The common hub of FC and SC is Occipital_Sup_R.

**TABLE 3:** List of common predictive connectivity features for the prediction of laser pain threshold.

Feature set	Regions or connectivity
FC	Frontal_Sup_R- Caudate_R Frontal_Sup_Orb_L—Parietal_Sup_R Frontal_Mid_Orb_L—Parietal_Sup_R Frontal_Mid_Orb_R—Occipital_Sup_R Frontal_Inf_Oper_L—Frontal_Inf_Tri_R Frontal_Inf_Oper_L—Cingulum_Mid_R Frontal_Inf_Oper_L—Parietal_Inf_R Frontal_Inf_Oper_L—Precuneus_R Frontal_Inf_Oper_R—Parietal_Sup_R Frontal_Inf_Tri_L—Parietal_Inf_R Frontal_Inf_Tri_R—Parietal_Inf_R Rolandic_Oper_L—Cingulum_Mid_R Rolandic_Oper_R—Cingulum_Mid_R Olfactory_L—Lingual_R Frontal_Sup_Medial_R—Caudate_R Rectus_R—Calcarine_L Insula_L—Cingulum_Mid_R Insula_L—Cuneus_L Insula_L—Occipital_Inf_R Insula_R—Cingulum_Mid_L Insula_R—Cingulum_Mid_R Cingulum_Ant_R—Occipital_Sup_R Cingulum_Mid_R—Occipital_Sup_L Cingulum_Mid_R—Occipital_Sup_R Cingulum_Mid_R—Parietal_Sup_L Cingulum_Mid_R—Parietal_Sup_R Hippocampus_R—Lingual_L Hippocampus_R—Fusiform_L ParaHippocampal_L—Fusiform_L ParaHippocampal_R—Fusiform_L Calcarine_L—Postcentral_L Calcarine_L—Temporal_Pole_Mid_R Occipital_Mid_R—Caudate_L Occipital_Inf_L—Pallidum_R Frontal_Mid_R—Frontal_Inf_Oper_L
SC	Occipital_Inf_R—Lingual_R Fusiform_R—Occipital_Sup_R

## Discussion

In this study, we investigated the predictive capability of multi-features of multi-modal MRI data in the prediction of individual pain sensitivity, as measured by laser pain threshold. The results on 210 healthy subjects demonstrated that fMRI-DTI and regional-connectivity features are capable of accurately predicting an individual’s pain threshold. Importantly, the predictive capability of fusing fMRI-DTI and regional-connectivity features is significantly higher than that of using one type of feature from one imaging modality (i.e., ReHo, FA, FC, or SC). These results revealed rich information about individual pain sensitivity from the brain’s both structural and functional perspectives as well as from both regional and connectivity brain patterns.

### Fused Model Achieves Higher Performance

The fused model that uses fMRI-based ReHo and FC features and DTI-based FA and SC features has the best prediction performance because (1) it uses both regional and connectivity features, and (2) it uses two imaging modalities.

First, the prediction models (i.e., fMRI Model, DTI Model, or Fused Model) which fused the regional features (i.e., ReHo and FA) and brain connectivity features (i.e., FC and SC) outperformed the prediction models which only used one type feature, suggesting that multi-type imaging features embrace richer information than single-type features in the prediction of pain sensitivity. The regional functional feature, ReHo, which reflects the spontaneous brain activity observed in specific regions, serves an important functional role in the efficacy of neural systems. Also, regional structural characteristics like FA reflect the changes in microstructure (Basser and Pierpaoli, 2011). Actually, several previous studies (Erpelding et al., 2012; Emerson et al., 2014; Rogachov et al., 2016; Geisler et al., 2021) have demonstrated the relationship between regional functional signal or structural characteristics and individual pain sensitivity. However, as pain is a complex experience related to a wide network of brain regions, it is also important to explore the relationship between individual pain sensitivity and brain connectivity which reflect the communication between distinct regions. Combining the information of both regional measurement and brain connectivity gives us a more complete understanding of the brain mechanisms underlying pain sensitivity, which may also be the reason for the improvement of the prediction performance after fusion. We will further discuss these predictive regional and connectivity features later in this section.

Second, we found that models based on both fMRI and DTI features are more predictive than those used single modality only, which implies these two MRI modalities contribute to the determination of pain sensitivity from different perspectives. But the predictive power of fused models is not simply equal to the sum of the power of the related prediction models which only used one MRI modality. This is expected because a wealth of research (Gu et al., 2015; Tang and Bassett, 2018) has shown that white matter microstructure links discreet brain areas and thus regulates brain function. In another word, DTI and fMRI features are correlated because of the brain’s structure-function coupling. Therefore, the information provided by fMRI and DTI, on the one hand, complements to each other, while on the other hand, overlaps to some extent. Although little effort has been made on utilizing the multimodal neuroimaging data (fMRI and DTI) for predicting individual pain sensitivity, several studies demonstrated the advantage of integrating DTI and fMRI in the field of cognitive neuroscience and psychiatry (Goble et al., 2012; Sugranyes et al., 2012). For example, Xiao et al. (2021) combined regional features extracted from the whole brain in three modalities (fMRI data, T1-weighted data, and DWI data), achieving a good performance in predicting individual visual working memory capacity.

### Functional Magnetic Resonance Imaging Features Predictive of Pain Sensitivity

Our results indicated some relations between the common predictive fMRI regional features and FC features. We found that the selected fMRI regional features are mainly located in the precuneus, insula, and calcarine. As identified in the previous studies, precuneus plays an important role in pain processing (Zhang et al., 2020), possibly with different mechanisms. Precuneus is engaged in continuous information gathering and representation of the self and the external world (co-perception), as well as in the assessment of self-relevant sensations (Johnson et al., 2006), both of which are important aspects of the pain experience. Also, the precuneus is a core constituent of the default-mode network (DMN) (Utevsky et al., 2014), of which the alterations have been well documented to be related to pain progression. In addition, Goffaux et al. (2014) found that pain sensitivity in healthy adults was closely tied to pain-evoked responses in the contra-lateral precuneus, which was similar to our study. Insula is a part of cortical regions that are related to the affective/motivational aspect of pain (Greenspan et al., 1999; Duerden and Albanese, 2013), and it is also important in the prediction of pain sensitivity.

As for common predictive FC features, a large number of prefrontal-parietal and insula-cingulate connectivity features were identified. Among these connections, we can easily find that some hubs, such as mid-cingulate cortex and insula, were also identified as common predictive regional results, which further showed the important roles of mid-cingulate cortex and insula in the determination of pain sensitivity. A study (Hsiao et al., 2020) has mentioned that pain sensitivity in healthy individuals is associated with the FC in pain-related cortical regions such as the insula. Beyond that, connections between prefrontal cortex and parietal lobe were also found to be the most important predictive connections. This finding is similar to previous studies (Tu et al., 2019), which found the frontal-parietal networks are useful in predicting an individual’s pain threshold at both with-session and between-session levels.

### Diffusion Tensor Imaging Features Predictive of Pain Sensitivity

For FA, the feature analysis demonstrated the FA features in Occipital_Inf_R, Temporal_Inf_R, Calcarine_R, Precuneus_R, Insula, and Lingual_R are useful for the prediction of laser pain threshold. Importantly, we could find that insula, precuneus, and calcarine are the common predictive regions identified from both fMRI regional features and DTI structural features. Therefore, these results do not only reflect the consistency of structure and function of the brain, but also confirm the key roles of these regions in the determination of pain sensitivity. For SC, occipital-occipital and occipital-temporal connections are predictive for the prediction of pain sensitivity. Actually, till now, only a few studies focused on the relationship between individual pain sensitivity and structural properties of white matter and the findings in these studies are inconsistent. Previous studies suggest that white matter properties are distinct between pain conditions (Mansour et al., 2013; Michels et al., 2017). For example, a DTI study found a negative correlation between FA and migraine duration in the mid-insula and a positive correlation between left mid-insula FA and pain catastrophizing (Mathur et al., 2016). Also, studies have provided evidence that white matter integrity within and between regions of the descending pain modulatory network is critically linked with the individual ability for endogenous pain control (Stein et al., 2012). Our study did not find many predictive DTI SC features in healthy individuals, which may imply that the white matter connectivity is mainly related to pain conditions of chronic patients but not closely related to healthy individuals’ pain sensitivity.

### Limitations and Future Work

Some limitations of the present study are mentioned here. First, the cerebellum was not included in the feature analysis because a proportion of participants had incomplete coverage of the cerebellum. Previous studies have suggested that the cerebellum has a role in pain and nociceptive processing (Moulton et al., 2010; Tu et al., 2019), so connectivity between the cerebellum and other regions may also be predictive of pain thresholds. Second, AAL atlas was used in our study to extract features in both the fMRI and DTI data. In fact, for each modality, there are more elaborate atlas options. To better compare the features between two modalities’ data, we finally chose commonly used AAL atlas to unify the atlas. Third, to some extent, the regional results showed lateralization to the right, which may be influenced by the location of the stimulus. To better validate the hypothesis, the measurement of pain sensitivity can be carried out on both left and right hands/legs. Moreover, pain sensitivity can be measured in many ways. In addition to the pain threshold used in our study, pain tolerance threshold and pain intensity can also be used to assess pain sensitivity. Meanwhile, different painful stimulus could also be used in pain measurement. To better describe subjects’ pain sensitivity, different pain measurements should be considered in the future studies. Fourth, the subjects recruited in this study were all young adults, but pain sensitivity and brain structure/function may vary across different ages. Several studies (Cole et al., 2010; El Tumi et al., 2017) have demonstrated that pain sensitivity varies with age. To better study the stability of pain sensitivity and understand the mechanism of pain sensitivity, it will be better to recruit a cohort with a more widely spectrum of ages in further studies. Finally, our finding would be helpful in understanding pain sensitivity in both structural and functional perspectives. However, the correlation between fMRI and DTI features and the underlying mechanism about how these multimodality features contribute together to affect the individual pain sensitivity remain unclear. Previous studies (Warbrick et al., 2017) have revealed that there is a relationship between fMRI features and DTI features and the relationship is task- and region-dependent. In our study, the relationship between the brain’s function and structure in these overlapping regions and how they work together to decide one’s pain sensitivity need to be confirmed by further studies. One possibility is that, a brain region’s function is (at least partially) determined by its structural characteristics and the brain function reflects complex multisynaptic interactions in structural networks (Suárez et al., 2020).

## Conclusion

In summary, we combined multi-features from multi-modal MRI data of healthy participants to investigate individual pain sensitivity and found that fusing functional and structural features as well as fusing regional and connectivity features can predict the individual pain threshold more accurately. Moreover, we identified several predictive features to individual pain sensitivity from both functional and structural perspectives as well as regional and connectivity perspectives. This study provides valuable information regarding how the brain’s structure, function, and connectivity interact and synergize in the determination of an individual’s pain sensitivity.

## Data Availability Statement

The orginal contributions presented in the study are included in the article/Supplementary Material, further inquires can be directed to the corresponding author/s.

## Ethics Statement

The studies involving human participants were reviewed and approved by the Ethics Committee of the Institute of Psychology, Chinese Academy Sciences, the Ethics Committee of Liaoning Normal University, and the Ethics Committee of Shenzhen University.

## Author Contributions

RZ, LL, and ZZ contributed to the construction of the study hypothesis. GH and ZL collected the data. RZ analyzed the data. RZ, LL, LZ, and ZZ discussed the results and wrote the manuscript. All authors contributed to the article and approved the submitted version.

## Conflict of Interest

The authors declare that the research was conducted in the absence of any commercial or financial relationships that could be construed as a potential conflict of interest.

## Publisher’s Note

All claims expressed in this article are solely those of the authors and do not necessarily represent those of their affiliated organizations, or those of the publisher, the editors and the reviewers. Any product that may be evaluated in this article, or claim that may be made by its manufacturer, is not guaranteed or endorsed by the publisher.

## Footnotes

1
http://rfmri.org/dpabi/

2
http://www.fil.ion.ucl.ac.uk/spm

3
http://www.nitrc.org/projects/panda

4
https://bioimagesuiteweb.github.io/webapp/connviewer.html
