# Supplementary material for: Combining Regional and Connectivity Metrics of Functional Magnetic Resonance Imaging and Diffusion Tensor Imaging for Individualized Prediction of Pain Sensitivity
Source: Front Mol Neurosci. 2022 Mar 15;15:844146. doi: 10.3389/fnmol.2022.844146 (PMC8965585; doi:10.3389/fnmol.2022.844146)
Supplement: Supplementary file 1 [file Data_Sheet_1.PDF]

Supplemental Materials

According to Supplementary Table 1, we compared the prediction performance of a series of models based on fMRI features (ReHo or FC) using different machine algorithms and different feature selection thresholds (P<0.05; P<0.01; P<0.001). According to the prediction performance (priority is given to the correlation coefficient), we selected the best model as the basic model for the following model fusion. Specifically, the best ReHo model finally used 0.001 as the feature selection threshold and SVR-Linear algorithm. FC model used 0.001 and SVR-Linear.

Supplementary Table 1. The prediction performance of a series of prediction models using different machine learning algorithms and different feature selection thresholds (fMRI)

Feature set	Method	MAE (mean±std)	PCC (R)
ReHo	SVR_RBF	0.41±0.33	0.13
	SVR_Linear	0.43±0.35	0.10
	PLSR	0.45±0.36	0.07
	RF	0.40±0.32	0.19
ReHo_05 (P<0.05)	SVR_RBF	0.42±0.34	0.18
	SVR_Linear	0.48±0.37	0.10
	PLSR	0.47±0.36	0.03
	RF	0.47±0.36	0.20
ReHo_01 (P<0.01)	SVR_RBF	0.43±0.33	0.18
	SVR_Linear	0.54±0.40	0.08
	PLSR	0.46±0.36	0.10
	RF	0.41±0.33	0.15
ReHo_001 (P<0.001)	SVR_RBF	0.42±0.33	0.20
	**SVR_Linear**	**0.42±0.33**	**0.30**
	PLSR	0.44±0.35	0.15
	RF	0.41±0.33	0.18
FC_all	SVR_RBF	0.42±0.32	0.09
	SVR_Linear	0.47±0.34	0.15
	PLSR	0.47±0.36	0.04
	RF	0.42±0.31	0.12
FC_05 (P<0.05)	SVR_RBF	0.42±0.31	0.18
	SVR_Linear	0.57±0.42	0.18
	PLSR	0.44±0.32	0.22
	RF	0.42±0.31	0.20

FC_01	SVR_RBF	0.43±0.31	0.19
(P<0.01)	SVR_Linear	0.55±0.45	0.07
	PLSR	0.44±0.32	0.22
	RF	0.42±0.30	0.21
FC_001	SVR_RBF	0.43±0.34	0.16
(P<0.001)	**SVR_Linear**	**0.43±0.32**	**0.23**
	PLSR	0.42±0.32	0.21
	RF	0.45±0.33	0.09

According to Supplementary Table 2, we compared the prediction performance of a series of models based on DTI features (FA or SC) using different machine algorithms and different feature selection thresholds (P<0.05; P<0.01; P<0.001). According to the prediction performance (priority is given to the correlation coefficient), we selected the best model as the basic model for the following model fusion. Specifically, the best FA model finally used 0.01 as the feature selection threshold and SVR-RBF algorithm. SC model used 0.001 and SVR-RBF.

Supplementary Table 2. The prediction performance of a series of prediction models using different machine learning algorithms and different feature selection thresholds (DTI)

Feature set	Method	MAE (mean±std)	PCC (R)
FA	SVR_RBF	0.41±0.33	0.14
	SVR_Linear	0.41±0.33	0.15
	PLSR	0.43±0.34	0.07
	RF	0.41±0.32	0.05
FA_05	SVR_RBF	0.41±0.33	0.13
	SVR_Linear	0.41±0.33	0.14
	PLSR	0.42±0.33	0.10
	RF	0.42±0.32	0.06
FA _01	**SVR_RBF**	**0.39±0.29**	**0.35**
	SVR_Linear	0.40±0.30	0.30
	PLSR	0.42±0.35	0.28
	RF	0.41±0.32	0.18
FA _001	SVR_RBF	0.48±0.35	-0.05
	SVR_Linear	0.48±0.34	-0.02
	PLSR	0.51±0.36	-0.08
	RF	0.43±0.34	-0.02
SC_all	SVR_RBF	0.43±0.32	-0.14
	SVR_Linear	0.42±0.32	-0.06
	PLSR	0.48±0.38	-0.15
	RF	0.42±0.33	0.01
SC_05	SVR_RBF	0.44±0.35	-0.05
	SVR_Linear	0.42±0.32	-0.15
	PLSR	0.55±0.44	-0.31
	RF	0.43±0.35	-0.11
SC_01	SVR_RBF	0.49±0.36	-0.36
	SVR_Linear	0.42±0.31	-0.24
	PLSR	0.53±0.43	-0.26

	RF	0.47±0.36	-0.20
SC_001	**SVR_RBF**	**0.40±0.30**	**0.30**
	SVR_Linear	0.42±0.34	0.17
	PLSR	0.42±0.33	0.15
	RF	0.41±0.32	0.30
